# Detection of Cause-Effect Relations Based on Information Granulation and Transfer Entropy

**DOI:** 10.3390/e24020212

**Published:** 2022-01-28

**Authors:** Xiangxiang Zhang, Wenkai Hu, Fan Yang

**Affiliations:** 1 School of Automation, China University of Geosciences, Wuhan 430074, China; zhangxiang2020@foxmail.com; 2 Hubei Key Laboratory of Advanced Control and Intelligent Automation for Complex Systems, Wuhan 430074, China; 3 Engineering Research Center of Intelligent Technology for Geo-Exploration, Ministry of Education, Wuhan 430074, China; 4 Beijing National Research Center for Information Science and Technology, Department of Automation, Tsinghua University, Beijing 100084, China; yangfan@tsinghua.edu.cn

**Keywords:** transfer entropy, information granulation, causality, root cause, oscillation

## Abstract

Causality inference is a process to infer Cause-Effect relations between variables in, typically, complex systems, and it is commonly used for root cause analysis in large-scale process industries. Transfer entropy (TE), as a non-parametric causality inference method, is an effective method to detect Cause-Effect relations in both linear and nonlinear processes. However, a major drawback of transfer entropy lies in the high computational complexity, which hinders its real application, especially in systems that have high requirements for real-time estimation. Motivated by such a problem, this study proposes an improved method for causality inference based on transfer entropy and information granulation. The calculation of transfer entropy is improved with a new framework that integrates the information granulation as a critical preceding step; moreover, a window-length determination method is proposed based on delay estimation, so as to conduct appropriate data compression using information granulation. The effectiveness of the proposed method is demonstrated by both a numerical example and an industrial case, with a two-tank simulation model. As shown by the results, the proposed method can reduce the computational complexity significantly while holding a strong capability for accurate casuality detection.

## 1. Introduction

In a complex large-scale process system, components and variables are interconnected through material flows and information flows. Once a fault occurs, it may easily propagate among units and cause negative impacts in broader areas, which may lead to serious consequences and compromise process safety. Therefore, it is important to detect and locate the root causes of faults as early as possible. Causality inference is a process to infer Cause-Effect relations between variables, typically in complex systems, and it is commonly used for root cause analysis in large-scale process industries. A variety of causality analysis techniques have been developed and shown to be effective for root cause diagnosis [1].

Existing techniques for causality inference can be generally divided into two types, namely, process knowledge-based methods and data-driven methods [1]. The former obtains connectivity and causality from prior knowledge, such as process topology and first-principle models, and convert the results into computer accessible formats, such as the adjacency matrix [2] and signed directed graph [3]. The latter captures Cause-Effect relations from sufficient process data; commonly used techniques include cross-correlation analysis (CCA) [4], granger causality analysis (GCA) [5], transfer entropy (TE) [6], and Bayesian networks (BN) [7,8]. References [9,10,11] compared the strengths and weaknesses of these techniques and also proposed suitable situations for their applications. In any case, to achieve better performance in root diagnosis, especially when abnormal situations are associated with unknown faults or multiple faults, integrated methods that combine process data analysis with process knowledge extraction were proposed in [12] and demonstrated to be quite effective.

Among the data-driven causality inference methods, transfer entropy (TE) provides an information-theoretic method for causality measurement that is suitable for both linear and nonlinear processes. TE was firstly proposed by Schreiber as a measure for information transfer [6]. As for whether TE measures causal relationships, there exists controversy. References, such as [13,14], discussed the distinctions between information transfer and causal effects. According to [14], information flow is a primary tool to establish the presence of causal relations, for where this is not possible, the complete transfer entropy is an alternate inference technique. As TE can effectively distinguish driving and responding elements and detect asymmetry in the interaction of subsystems, it has been widely studied and used for causality inference.

Reference [15] utilized TE to infer causal relations for the identification of the propagation direction of disturbances. In [16], kernel principal component regression and transfer entropy were combined to conduct root cause diagnosis. In addition, some variants or improvements have been proposed to extend TE. For instance, in order to distinguish the direct or indirect causal relations, partial transfer entropy [17] and direct transfer entropy [18] were developed. Reference [19] proposed the transfer zero-entropy for causality analysis based on the zero-entropy and zero-information without assuming a probability space. Additionally, symbolic transfer entropy [20] and trend transfer entropy [21] extended the TE to symbols or trends of time series instead of original continuous values. The multiple-unit symbolic dynamics and transfer entropy were used to analyze the dynamic causal relationships in longitudinal data [22]. A symbolic dynamic-based normalized transfer entropy (SDNTE) was proposed for the root cause fault diagnosis of multivariate nonlinear processes [23].

In the field of alarm root cause analysis, TE was adpated to analyze Cause-Effect relations among binary-valued alarm variables [24]; moreover, a Bayesian network based on active dynamic transfer entropy (ADTE) was proposed to establish an accurate alarm propagation network during an alarm flood [25]. For oscillation diagnosis, a workflow using TE was proposed to provide a robust procedure for accurately identifying the oscillation propagation path [26]. In addition, TE and Granger causality were tested on an industrial case study of a plant-wide oscillation, and how to choose between the two methods in actual industrial applications was explained [11].

As shown by the extensive studies above, transfer entropy has become a prevalent and effective way of capturing Cause-Effect relations in complex systems. However, a major problem with TE lies in its high computational complexity, which prevents it from applications in many real systems, especially for real-time tasks such as online root cause diagnosis. According to [15,18,27], the computational complexity of TE is mainly restricted by the estimation of probability density functions and the calculation of the transfer entropy in a high dimensional embedding space. Motivated by the above problem, this paper proposes an improved method for causality inference based on transfer entropy and information granulation. The calculation of transfer entropy is improved with a new framework that integrates the information granulation as a critical preceding step; moreover, a window-length determination method is proposed based on delay estimation, so as to conduct appropriate data compression using information granulation. The effectiveness of the proposed method is demonstrated by both a numerical example and an industrial case with a two-tank simulation model. As shown by the results, the proposed method can reduce the computational complexity significantly while holding a strong capability for accurate casuality detection.

The advantages of the proposed method lie in two aspects: (1) Compared to Cross-Correlation [4] and Granger Causality [5], which work only for linear causal relations, the proposed method inherits the advantage of TE in capturing non-linear causal relations and, thus, can be applied to broader fields. (2) Compared to traditional TE methods [14,15,16,17,18], the proposed method has much higher computational efficiency on account of the discretization and information granulation as preprocessing steps, and thus, it can be used for real-time tasks (e.g., online root cause diagnosis) that are sensitive to calculation time.

The rest of this paper is organized as follows. Section 2 presents the preliminaries of TE and analyzes the computational complexity problem. Section 3 proposes the improved calculation of TE. Section 4 provides case studies to demonstrate the effectiveness of the proposed method, followed by concluding remarks in Section 5.

## 2. Preliminaries on Transfer Entropy

Measures for quantifying dependency for bivariate or multivariate time series include the correlation coefficient, cross-correlation, and mutual information [28]. Mutual information quantifies the dependency from the joint probability density function of two random variables. It measures the reduction of uncertainty of a random variable based on the knowledge of a second variable, but cannot measure its directionality or causality. The information theory measure of transfer entropy proposed in [6] takes the concept of mutual information a step further. Transfer entropy is an asymmetric measurement method based on information theory. By calculating the conditional probability function and designing a reasonable directionality measure, the causal topology is constructed to facilitate root cause diagnosis and propagation path identification.

Based on the concept of information theory, the measure of transfer entropy proposed by Schreiber [6] extracts the amount of information transferred from variable *x* to *y* as follows:

(1) $$T_{x\to y}=\sum p\left(y_{i+h},y_{i}^{\left(k\right)},x_{i}^{\left(l\right)}\right)\cdot \log\frac{p\left(y_{i+h}|y_{i}^{\left(k\right)},x_{i}^{\left(l\right)}\right)}{p\left(y_{i+h}|y_{i}^{\left(k\right)}\right)},$$

where p() indicates the joint or conditional probability density function (PDF); *k*, *l* are the orders of variables *y*, *x*; *h* is the prediction horizon; $x_{i}^{\left(l\right)}=\left[x_{i},x_{i-\tau },\ldots ,x_{i-(l-1)\tau }\right]$ and $y_{i}^{\left(k\right)}=\left[y_{i},y_{i-\tau },\ldots ,y_{i-(k-1)\tau }\right]$; τ are the sampling periods.

In order to remove the indirect causality caused by the intermediate variables or the false causality caused by the common variables, the direct transfer entropy (DTE) proposed in [18] can be calculated, i.e.,

(2) $$D_{x\to y|z}=\sum p\left(y_{i+h},y_{i}^{\left(k\right)},z_{i}^{\left(m\right)},x_{i}^{\left(l\right)}\right)\cdot \log\frac{p\left(y_{i+h}|y_{i}^{\left(k\right)},z_{i}^{\left(m\right)},x_{i}^{\left(l\right)}\right)}{p\left(y_{i+h}|y_{i}^{\left(k\right)},z_{i}^{\left(m\right)}\right)},$$

where *z* represents the intermediate variable, *m* denotes the order of the intermediate variable *z*, and $z_{i}^{\left(m\right)}=\left[z_{i},z_{i-\tau },\ldots ,z_{i-(m-1)\tau }\right]$.

Transfer entropy is effective in measuring the causality for both linear and nonlinear processes. A major problem hindering the application of TE lies in its high computational complexity, which is mainly contributed to by the estimation of probability density functions (PDFs) and the calculation of TE in a high-dimensional embedding space. In this study, the required data type is continuous valued time series, and thus, PDF estimation is a mandatory step. There are many methods for PDF estimation, such as plug-in estimators, kernel density estimators, and nearest-neighbor-based estimators. The run time of estimating TE may vary depending on the estimator chosen. The improvement of PDF estimators is not investigated here; as presented in many related studies [15,29], the commonly used kernel density estimator is exploited for PDF estimation. The focus of this paper is to investigate the calculation of TE in a high-dimensional embedding space, and to put forward a corresponding solution to reduce the computational complexity.

The total computational complexities for TE and DTE are $O(N^{2}{\left(k+l\right)}^{2})$ and $O(N^{2}{\left(k+l+m\right)}^{2})$, respectively [18], where *N* is the sample size. Obviously, the computation complexity is mainly decided by two factors, namely, the sample size and the order. In view of this, improving the efficiency of TE needs to address two problems: (1) How to reduce the sample size processed by TE, and (2) how to reduce the orders of the cause and effect variables. The key is that it should guarantee the accuracy in causality inference while addressing the two problems of TE. Hereby, this work improves the transfer entropy with a new framework that integrates the information granulation as a critical preceding step, which conducts data compression and, thus, uses information granules in TE calculation. The details of the proposed method are presented in the next section.

## 3. The Proposed Method

This section presents the improved TE based on information granulation. Specifically, this section provides the framework of the proposed method, the data abstraction via information granulation, the calculation of information granulation-based TE, and the determination of the granulation window size.

### 3.1. The Framework

**Figure 1:**
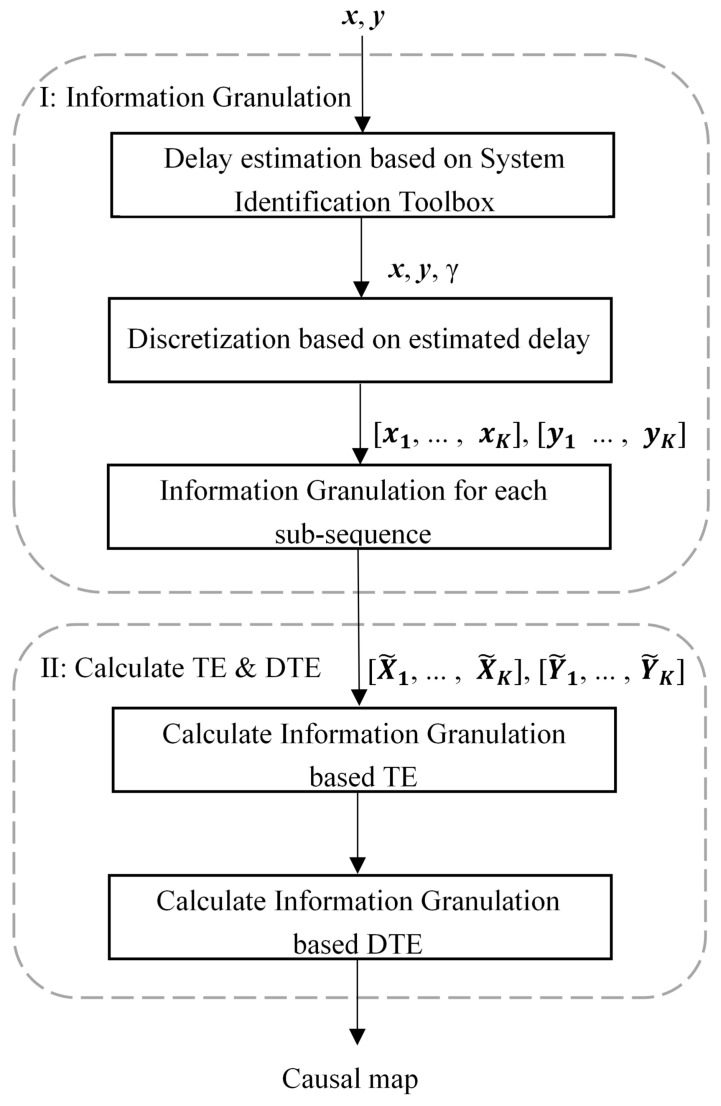
The framework diagram of the proposed method.

Given a pair of time series x and y, the objective is to infer their causal relation using TE. As discussed in Section 2, to improve the efficiency of TE, the effective solution is to reduce the sample size processed by TE and to reduce the orders of cause-and-effect variables in the calculation of TE. Accordingly, this work proposes the following framework, which integrates TE and information granulation for causality inference. A diagram is shown in Figure 1 to present the framework of the proposed method.

First, to reduce the sample size, it should compress the time series and extract a shorter sequence consisting of representative values in consecutive time windows. However, it is also noteworthy that the length of the window size is a critical parameter influencing the final analysis result. If the time series is compressed too much with a large window size, the computation is reduced, and the price is that useful information might be lost and, thus, lead to erroneous conclusions in causality inferences.

Second, to reduce the orders of the cause-and-effect variables, it only needs to use the first-order TE, where the orders of both cause and effect variables are ones. However, applying the first-order TE requires that the delay between two variables should be 1; otherwise, it might give wrong causal relations. Therefore, properly compressing the data in the previous step is critical.

The information granule obtained after granulation reduces the scale of the original data, and the amplitude also changes to a certain extent. It has been learned from previous work that this may lead to a biased conclusion. For example, Refs. [30,31,32] discussed the influence of sampling rate and time scale on causal inference through the test of data such as EEG signals, and explained that this might change the causal relations. In addition, the impact of data filtering and amplitude changes on causal analysis was investigated in [29,33,34,35]. An unreasonable sampling rate and a changed series will lead to false causality. Motivated by the investigation in these previous studies, a systematic method of information granulation with delay estimation is proposed for data processing. The comparison in the case study in Section 4 demonstrates the rationality of the method, i.e., given a proper estimated window size, the proposed method will ensure the correctness of the detected causality.

### 3.2. Data Abstraction via Information Granulation

**Figure 2:**
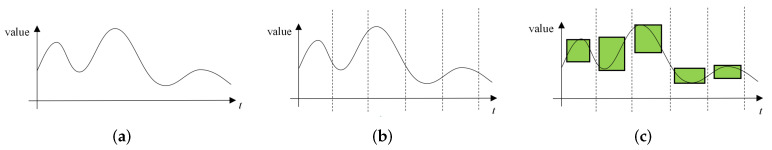
The schematic of the information granulation process. (**a**) Original time series; (**b**) Discretization; (**c**) Information granulation for each subsequence.

The information granulation of time series is the basis for compressing the scale of time series data and using the compressed data for subsequent time series analysis, interpretation, and modeling. The information granulation of time series specifically includes two main steps (shown in Figure 2):

- Discretization: Given a time series $x=[x_{1},x_{2},\ldots ,x_{N}]$, *K* non-overlapping subsequences $x_{1},x_{2},\ldots ,x_{K}$ are obtained by discretization. The data in each subsequence can be accurately described by a simple model;
- Information granulation for each subsequence: The information granulation operation is performed on subsequence $x_{k}=\left[x_{1},x_{2},\ldots ,x_{w}\right]$ (where k=1,2,⋯,K, and *w* indicate the window length), so as to form a time-related information granule ${\tilde{X}}_{k}$ that represents the data characteristics of this subsequence.

After the above two steps, the original time series is converted into the corresponding granular time series $\tilde{X}=\left[{\tilde{X}}_{1},{\tilde{X}}_{2},\ldots ,{\tilde{X}}_{K}\right]$, where ${\tilde{X}}_{k}=\left[{\tilde{X}}_{k}^{1},{\tilde{X}}_{k}^{2},{\tilde{X}}_{k}^{3}\right]$ is the *k*th information granule.

In the past, various IG methods were proposed, such as the fuzzy set-based IG [36,37], clustering-based IG [36] and intelligent optimization-based IG [38,39]. Among them, the amount of data contained in clustering-based IG is limited, and information loss is large [36]; the intelligent optimization-based IG is computationally time-consuming, which conflicts with the goal of reducing computational complexity in this study. Therefore, the fuzzy set-based IG is adopted since it makes use of more effective data [36] and has a fast calculation speed.

Zadeh [40] gave a general definition of fuzzy information granules. It is represented, using fuzzy sets, as:

(3) g≜(xisG)isϱ,

where *x* is a variable in the universe *U*; *G* is a convex fuzzy set of *U*, described by a membership function μG; ϱ is the probability. The core issue of the information granulation method based on fuzzy sets is to determine a membership function A=μG. The representation of information granules produced by the fuzzy set-based method is closely related to $x_{i}$. The triangular membership function is given as:

(4) $$A\left(x\right)=\left\{\begin{array}{c} \;0,x<a,\\ \;\frac{x-a}{c-a},a\leq x\leq c,\\ \;\frac{b-x}{b-c},c<x\leq b,\\ 0,x>b, \end{array}\right.$$

where *a*, *c*, and *b* are the parameters of the triangular membership function.

The information granulation method based on fuzzy sets developed in [41] is employed. A good granulation process should satisfy two requirements: (i) the raw data are fully expressed by information granules; (ii) information granules should become specific enough. To meet these requirements, a function $Q_{A}\left(x\right)$ with respect to the membership function A(x) is constructed to describe the performance of the granulation process, i.e.,

(5) $$Q_{A}=\frac{M_{A}}{N_{A}},$$

where $M_{A}=\sum_{k=1}^{w}A\left(x_{k}\right)$, and maximizing $M_{A}$ can meet the requirement (i); $N_{A}=\mathrm{measure}(\mathrm{support}\left(A\right))$, and minimizing $N_{A}$ can meet the requirement (ii). Apparently, in light of the aforementioned requirements, $Q_{A}$ has to be maximized.

Then, the fuzzy information granules can be expressed as $\tilde{X}=\left[{\tilde{X}}^{1},{\tilde{X}}^{2},{\tilde{X}}^{3}\right]$, where ${\tilde{X}}^{1}$ and ${\tilde{X}}^{3}$ are the supports, and ${\tilde{X}}^{2}$ is the core. By calculating the three parameters *a*, *c*, and *b* of the triangle membership function in Equation (4), the corresponding ${\tilde{X}}^{1},{\tilde{X}}^{2},{\tilde{X}}^{3}$ are obtained. The core of the information granule is calculated by:

(6) $${\tilde{X}}_{i}^{2}=\mathrm{med}\left(x_{1}^{i},x_{2}^{i},\cdots ,x_{w}^{i}\right),$$

which is the median of subsequence. According to [41], taking into account the triangular membership function, when $Q_{A}$ is maximum, ${\tilde{X}}^{1}$ and ${\tilde{X}}^{3}$ can be directly calculated by:

(7) $$\left\{\begin{array}{c} \;{\tilde{X}}_{i}^{1}=\frac{2}{\left[w/2\right]}\sum_{j=1}^{\left[w/2\right]}x_{j}^{i}-{\tilde{X}}_{i}^{2},\\ \;{\tilde{X}}_{i}^{3}=\frac{2}{w-[w/2]-d+1}\sum_{j=[w/2]+d}^{w}x_{j}^{i}-{\tilde{X}}_{i}^{2}, \end{array}\right.$$

where [w/2] denotes the largest integer not exceeding w/2(w≥2); $x_{j}^{i}$ represents the *j*-th sample in the *i*-th subsequence $\left[x_{1}^{i},x_{2}^{i},\cdots ,x_{w}^{i}\right]$. In addition, when *w* is an even number, d=1; otherwise, d=2. Through the above calculation, the granular time series is obtained for subsequent analysis.

**Remark** **1.** *Using granulation to process the original data, the granule with greatly reduced data length is obtained. The advantage of the granulation as a preprocessing step is that it cannot only reduce the size of data, but also suppress noises effectively. However, the granulation may reduce the amplitude resolution, alter the value of the TE estimates, and even change the direction of detected causal relation. This may happen when the granular time series does not hold the dynamics and the variational trend of the original data. The key lies in the selection of a proper window length in discretization. If only the window length in discretization is set properly, the granular time series can retain the dynamic characteristics of the original data and keep the main variational trend. If the window length is too small, the dynamics are retained but the data compression is not effective. By contrast, if the window length is too large, the granular data may lose the dynamics and lead to erroneous conclusions in causality inference. To achieve maximum data compression and also retain the dynamics, this work proposes taking the delay between two time series as the window size. After compressing the data via granulation, the dynamics and the variational trend are retained in the one-sample history. The casual relation reflected by such one-sample history can be measured by the first-order TE. Thus, such a preprocessing approach will not influence the TE estimates too much, and can guarantee that detected causal relation is consistent with the one detected from original data while making a much faster calculation. The discussion on the window length determination is presented in Section 3.4. The validity of the approach is verified by extensive simulations. Further, in case studies, the proposed method was compared with the traditional TE, and the causal relations were found to be consistent.*

### 3.3. Calculation of the Information Granulation-Based Transfer Entropy

Given the granular time series $\tilde{X}=\left[{\tilde{X}}_{1},{\tilde{X}}_{2},\ldots ,{\tilde{X}}_{K}\right]$ and $\tilde{Y}=\left[{\tilde{Y}}_{1},{\tilde{Y}}_{2},\ldots ,{\tilde{Y}}_{K}\right]$, ${\tilde{X}}_{i}=\left[{\tilde{X}}_{i}^{1},{\tilde{X}}_{i}^{2},{\tilde{X}}_{i}^{3}\right]$ is the *i*th information granule. To avert the problem caused by information loss through information graduation, the calculation of TE exploits all the three items in the information granule and takes the average as the final TE result, which is supposed to ensure the reliability of the result.

Through proper information granulation, it can offset the effect of delay and reduce the delay between two variables to only 1 sample. Therefore, only the first-order situation needs to be considered when calculating TE here. That is, k=l=1 and h=τ=1. It should be noticed that delay embedding is usually used in causality inference so as to include the relevant past of the time series in the estimate of TE. References [42,43] provided systematic methods for finding appropriate embedding lengths. This is helpful for getting more accurate estimates of TE for causal relations reflected by more than one sample history. However, in this study, the information granulation needs to ensure that the data are compressed as much as possible, while the dynamics are still retained in the granular time series. Accordingly, the multi-sample history is compressed to a one-sample history, such that the delay between the granular time series is 1. Thus, the calculation of TE only needs to consider the first order, rather than high orders. Therefore, the formula for information granulation-based TE, from *x* to *y*, is given by:

(8) $${\tilde{T}}_{x\to y}^{j}=\sum p\left({\tilde{Y}}_{i+1}^{j},{\tilde{Y}}_{i}^{j},{\tilde{X}}_{i}^{j}\right)\cdot \log\frac{p\left({\tilde{Y}}_{i+1}^{j}|{\tilde{Y}}_{i}^{j},{\tilde{X}}_{i}^{j}\right)}{p\left({\tilde{Y}}_{i+1}^{j}|{\tilde{Y}}_{i}^{j}\right)},$$

where ${\tilde{Y}}_{i}^{j}$ or ${\tilde{X}}_{i}^{j}\left(j=1,2,3\right)$ represent the *i*th sample in the *j*th dimension of the information granule, which is obtained by information granulation for *y* or *x*. The kernel density estimator is applied in this paper to estimate the PDFs. The three dimensions are used to calculate TE using Equation (8), and the average of the three results is taken as the final result.

In order to remove the indirect causality caused by the intermediate variables or the false causality caused by the common variables, the DTE can be calculated. From the causal network detected by TE, some causal relations could be indirect through the influence of intermediate or confounding variables. For instance, given a pair of variables *x* and *y* holding a causal relation, if there is a third variable *z* making a triangle network (i.e., *z* is the intermediate or confounding variable holding causal rations with both *x* and *y*), it is necessary to detect whether the causal relation between *x* and *y* is direct, or indirect through a pathway from *z*. As a result, the calculation of DTE can simplify the causal network and obtain more accurate results.

The granular time series of an intermediate variable *z* is obtained and denoted by $\tilde{Z}=\left[{\tilde{Z}}_{1},{\tilde{Z}}_{2},\ldots ,{\tilde{Z}}_{K}\right]$. Analogous to DTE [18], the information granulation-based DTE is defined as:

(9) $${\tilde{D}}_{x\to y|z}^{j}=\sum p\left({\tilde{Y}}_{i+1}^{j},{\tilde{Y}}_{i}^{j},{\tilde{Z}}_{i}^{j},{\tilde{X}}_{i}^{j}\right)\cdot \log\frac{p\left({\tilde{Y}}_{i+1}^{j}|{\tilde{Y}}_{i}^{j},{\tilde{Z}}_{i}^{j},{\tilde{X}}_{i}^{j}\right)}{p\left({\tilde{Y}}_{i+1}^{j}|{\tilde{Y}}_{i}^{j},{\tilde{Z}}_{i}^{j}\right)},$$

where ${\tilde{Z}}_{i}^{j}$ represents the *i*th sample in the *j*th dimension of the information granule of *z*. The three dimensions are used to calculate DTE using Equation (9), and the average of the three results is taken as the final result.

To determine whether a causal relation holds, it needs to compare the obtained TE with a threshold. An effective method to determine the threshold is the Monte Carlo method based on surrogate data. IG-based TEs are calculated using surrogate data that are generated randomly [44], and then their mean and standard deviations are obtained to acquire the threshold $S_{x\to y}$ [15,45]. By comparing ${\tilde{T}}_{x\to y}$ with the threshold $S_{x\to y}$, the causal relation between *x* and *y* is determined. If ${\tilde{T}}_{x\to y}\geq S_{x\to y}$, it indicates that there is a causal relation from *x* to *y*; otherwise, it suggests no causality from *x* to *y*. Analogously, IG-based DTEs are calculated using surrogate data, and then their mean and standard deviations are obtained to acquire the threshold $S_{x\to y|z}^{d}$. If ${\tilde{D}}_{x\to y|z}\geq S_{x\to y|z}^{d}$, there is a direct causal relationship from *x* to *y* based on *z*; otherwise, there is no direct causality from *x* to *y*.

Here, the computational complexities of TE and IG-based TE are compared. According to Section 2, for traditional TE, the computational complexity is $O(N^{2}{\left(k+l\right)}^{2})$. As for the IG-based TE, the computational complexity is $O\left({\left(N/w\right)}^{2}{\left(1+1\right)}^{2}\right)=O\left(4{\left(N/w\right)}^{2}\right)$, and *w* denotes the window length. Thus, it can be seen that the proposed method can greatly reduce the computational complexity for the TE calculation.

### 3.4. Determination of the Window Length by Delay Estimation

When performing information granulation on original data, there is a key parameter that needs to be discussed, namely, the window length *w* during discretization. If the window length *w* is too large or too small, the TE calculation result will be affected. Specifically, a large window length in information granulation can reduce the computational complexity, but may also lead to information loss and, thus, compromise the accuracy of causality detection. Therefore, a reasonable choice of window length is essential for correct causality analysis.

As discussed in Section 3.3, the first-order TE is used. Thus, the window length should be set to offset the delay between two time series in the original data, such that the delay between two compressed time series after information granulation is 1. Accordingly, this paper proposes determining the window length through delay estimation. It should be noticed that determining the window length by the delay between two time series *x* and *y* is based on an assumption that the history of the target time series *y* should be no more than the delay between *x* and *y*. Otherwise, if the assumption is violated, the relevant history of *y* might not be fully included in the estimate of the first-order TE and, thus, it may falsely estimate the TE, as indicated in [46,47].

Here, the system identification toolbox in MATLAB is used to estimate the delay between data [11]. The main procedures are as follows:

1. The original data of two variables *x* and *y* are normalized by z-sorce, i.e., $x^{*}=\frac{x-\mu }{\sigma }$, where $x^{*}$ denotes the normalized sample of *x*; μ and σ denote the mean and standard deviation, respectively;
2. Given the normalized data, the estimation is conducted based on a comparison of ARX models with a range of time delays, i.e., $y\left(t\right)+a_{1}y\left(t-1\right)+a_{2}y\left(t-2\right)=b_{1}x\left(t-\gamma \right)+b_{2}x\left(t-1-\gamma \right)+e\left(t\right)$, where γ denotes delay between *x* and *y*; $a_{1},a_{2},b_{1},b_{2}$ are the coefficients of the model; e(t) is white noise.

Through the above process, the delay between the two variables is obtained. Then, the window length in information granulation is assigned with the value of time delay so as to make the delay between granular time series be 1, such that the first-order TE in Section 3.3 is applicable. Next, an example is presented to illustrate the determination of window length through delay estimation.

**Example** **1.** *Given the relation between two nonlinearly correlated continuous random variables x and y as $y_{k+2}=1+2\left|10-\left(0.5x_{k}+0.2\sqrt{y_{k}}\right)\right|+v_{k}$, where $x_{k}=100sin\left(0.63t\right)+v$, the sampling time t is 0.01, v∼N(0,0.5), $v_{k}∼N\left(0,0.05\right)$, and y(0)=0.2. The simulation data of 3000 samples under stationary period are collected.*

*Using the above method, the time lag between x and y was obtained as 2, which was consistent with the actual value. Then, the data were abstracted through information granulation by taking the time lag as the window length. The IG-based TEs were calculated with the data in three dimensions of granular time series.*

**Figure 3:**
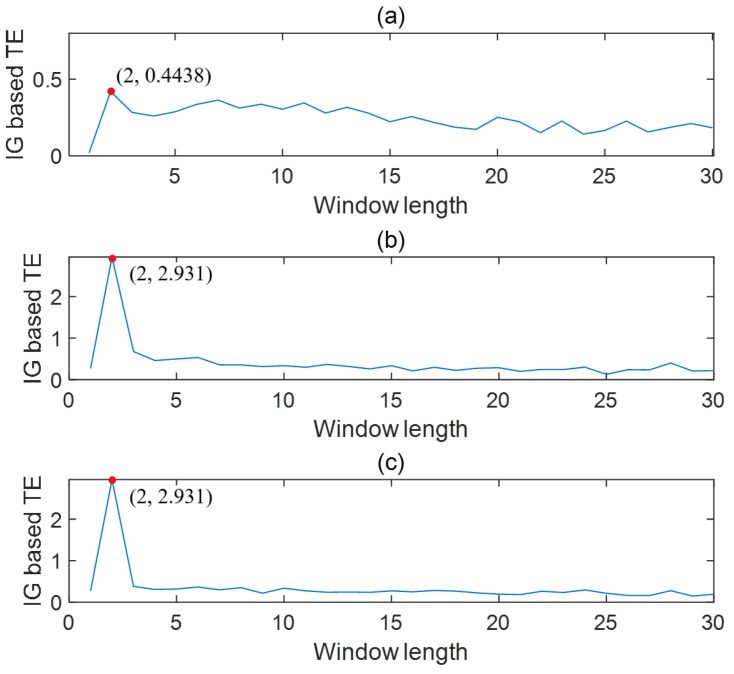
IG-based TE changes with the window length (from *x* to *y*). (**a**) shows the result calculated using ${\tilde{X}}^{1}$; (**b**) shows the result calculated using ${\tilde{X}}^{2}$; (**c**) shows the result calculated using ${\tilde{X}}^{3}$.

*To test how the window size of information granulation influences the TE calculation, a series of simulations were conducted by changing the value of the window size. Figure 3 presents the trends of IG-based TEs changing with the window length. It can be seen that the maximum TEs for three dimensions of the granular time series can be found at the point where the window length is equal to the delay, as indicated by the highlighted solid circles. Thus, it verifies the idea that determining the window length of information granulation can be based on the time delay between variables.*

*According to the result shown in Figure 3, the proposed IG-based TE correctly detected the causal relation when the window length was set to be the time delay. By contrast, the causal strength changed and erroneous conclusions were obtained if an inappropriate window length was used, as demonstrated by the very small calculated values of TE for other window lengths in Figure 3. Thus, the validity of the proposed approach was verified. The correct causal relations can be obtained as long as a proper window length is used in discretization. After compressing the data via granulation that takes the time delay as the discritzation window length, the casual relation is reflected by one sample history and can be measured by the first-order TE rather than a higher order TE. To demonstrate this, the values of TE with different orders were calculated for the same granular time series. The results are shown in Table 1. It can be seen that the value of TE stays high and does not change too much with the increasing of the orders. Thus, the first-order TE is enough to measure the causal relation given the properly compressed data.*

## 4. Case Studies

This section provides both a numerical example and an industrial case study to demonstrate the effectiveness of the proposed method.

### 4.1. Numerical Example

Assume three nonlinearly correlated continuous random variables x,y, and *z*, satisfying:

(10) $$\left\{\begin{array}{c} z_{k+3}=1+2\left|10-\left(0.5x_{k}+0.2\sqrt{z_{k}}\right)\right|+v_{k},\\ y_{k+2}=2{\left(z_{k}+2\right)}^{2}+10\sqrt{x_{k}}+{\tilde{v}}_{k}, \end{array}\right.$$

where $x_{k}=100\sin\left(0.63t\right)+v$, and the sampling time *t* is 0.01, v∼N(0,0.5), and $v_{k},{\tilde{v}}_{k}∼N\left(0,0.05\right)$. The simulation data of 3000 samples under stationary periods are collected.

Through delay estimation, the time lag between *x* and *y* was obtained as 2, that between *x* and *z* was 3, and that between *z* and *y* was 2. It can be seen from the formula that the obtained time lags are the same as the actual values. Then, the data were abstracted through information granulation by taking the time lags as the window lengths. The IG-based TEs were calculated with the data in three dimensions of granular time series.

**Figure 4:**
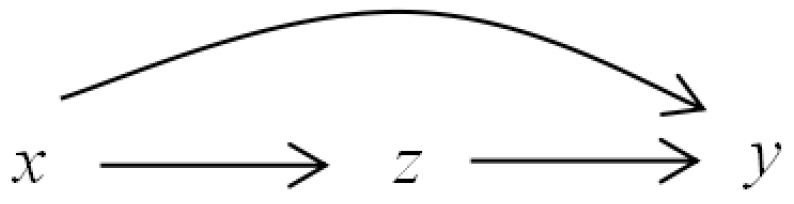
The information flow pathways for the numerical example.

The information granulation based-transfer entropies and the corresponding thresholds between each pair of *x*, *z*, and *y* are shown in Table 2. By comparing the TEs and the thresholds, it can be concluded that *x* causes *y*, *z* causes *y*, and *x* causes *z*, which is consistent with the relations in Equation (10). The information flow pathways for this numerical example are shown in Figure 4.

**Figure 5:**
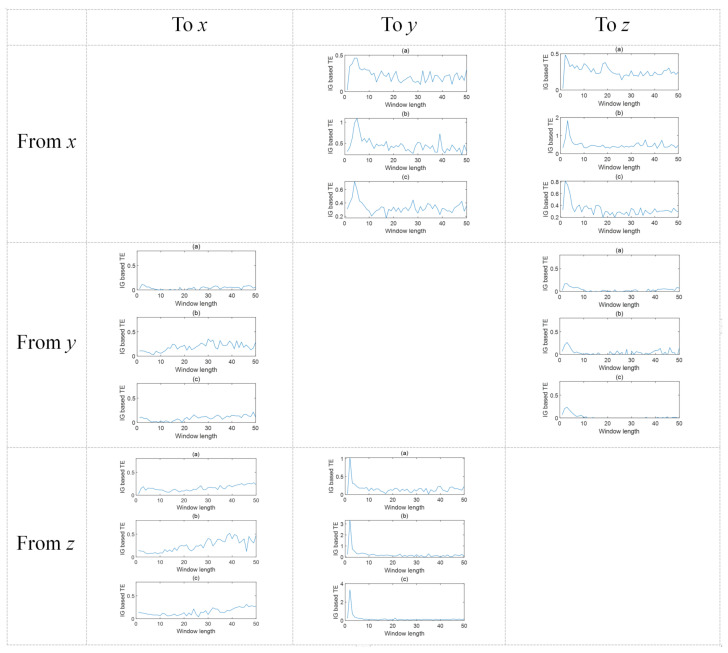
The trends of IG-based TEs under three different dimensions of the granular time series versus the window length. Subplots (**a**), (**b**), and (**c**) correspond to the results based on the lower support ${\tilde{X}}^{1}$, the core ${\tilde{X}}^{2}$, and the upper support ${\tilde{X}}^{3}$, respectively.

Figure 5 presents the IG-based TEs in three dimensions of the granular time series under different window lengths ranging from 0 to 50. It can be seen that the maximum values of the TEs can be found at where the window length is equal or nearly equal to the given time delay. This also verifies the feasibility of the proposed method. In addition, calculating the TEs in three dimensions can make the result more reliable and convincing. This is because, in some cases, when calculating TEs using different dimensions of the granular time series, the largest values of TEs might not always locate exactly at the window length equal to the time delay. Therefore, it is more reasonable to make use of the three dimensions and obtain an integrated value of TEs. The result in this example proves that the causal relations obtained using the proposed method are consistent with the actual case.

In order to compare the causality results obtained, the traditional TE method was also applied. The calculated TEs are in Table 3. By comparing Table 2 and Table 3, it can be observed that the detected causal relations are consistent using the proposed IG-based TE and the traditional TE. Thus, the proposed method ensures the accuracy of the detection of causal relations in this numerical case study.

Next, the calculation time of the proposed method was compared with the traditional TE. The TEs between the three variables were calculated. The total calculation time of each method is given in Table 4. Compared with the traditional TE, the calculation time is improved by 88.2% using the proposed method. Thus, the improvement of computational efficiency is significant.

### 4.2. Industrial Case Study

**Figure 6:**
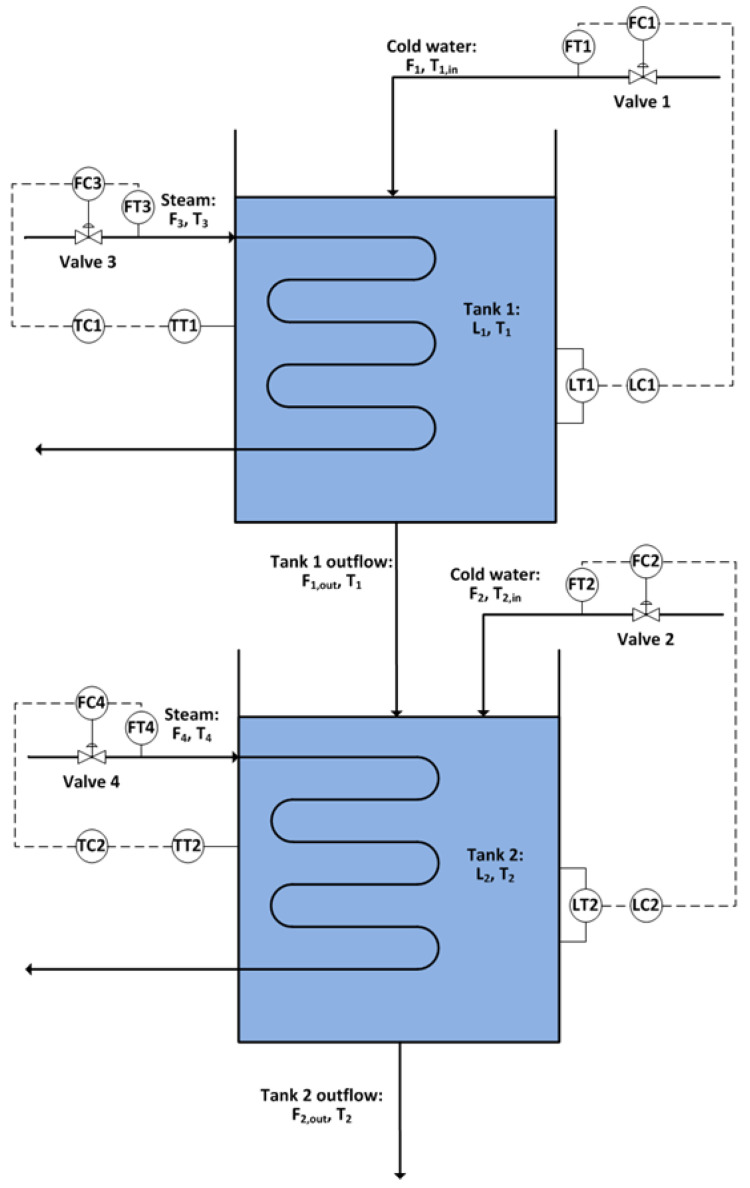
Diagram of the two-tank process.

The proposed method was applied to an industrial case for the root cause detection of plant oscillations. The fundamental model of the system was constructed in Simulink [11]. It has been used as a benchmark to test the process monitoring and causality inference methods [26,48,49]. Figure 6 shows a diagram of the process. There are two tanks in series with heat exchangers. Tank levels are controlled by the flow rates of cold water into the tanks, and tank temperatures are controlled by the steam flow rates through the heating coils.

**Figure 7:**
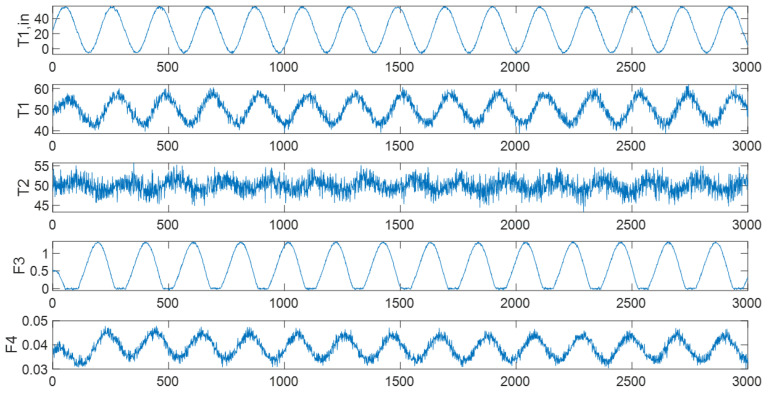
The time series of the original variables.

An oscillation was introduced in the cold water input temperature $T_{1,in}$. This oscillation would propagate through the process from $T_{1,in}$ by firstly affecting the first tank’s temperature $T_{1}$. The temperature controller would then change the steam flow rate $F_{3}$ to compensate. The controller was unable to fully reject the input disturbance and the second tank’s temperature $T_{2}$ would also be affected; the second tank’s temperature controller would then change the steam flow rate $F_{4}$ to compensate. By running the simulation, process signals were collected, and random noises were added to the signals. The original time series of the variables are shown in Figure 7.

**Figure 8:**
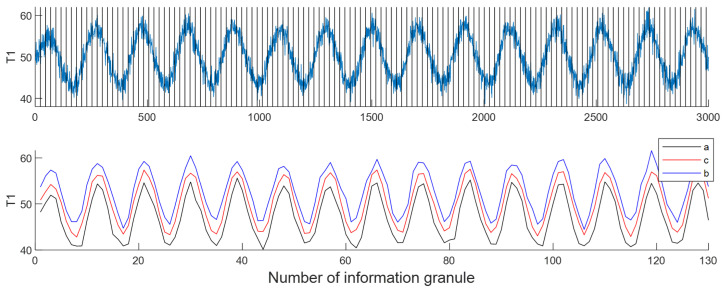
An example of information granulation for $T_{1}$.

First, calculate the time lag through delay estimation. Then, determine the window length based on the delay and perform the information granulation operation. Here, the delay between $T_{1,in}$ and $T_{1}$ is 23. Thus, the window length is set to 23. After performing the information granulation operation, the time series of information granulation of $T_{1,in}$ is shown in Figure 8.

**Figure 9:**
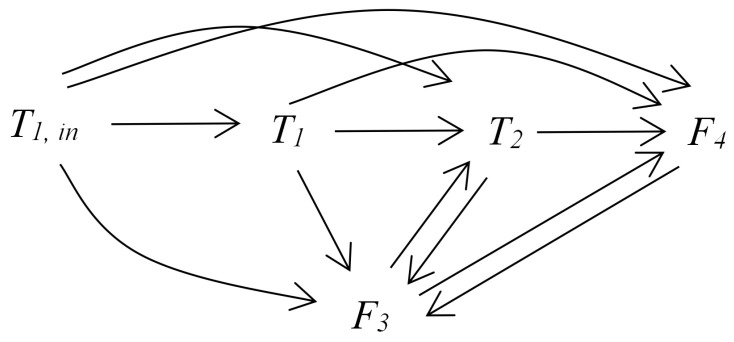
The information flow pathways for the two-tank system.

Then, the data of information granule ${\tilde{X}}_{i}$ are used to calculate TE. Using the proposed method, it can obtain the causal relations between variables according to Table 5. Figure 9 presents the causal map for the variables of the two-tank system.

In order to compare the causality results obtained, the traditional TE method was also applied. The calculated TEs are in Table 6. By comparing Table 5 and Table 6, it can be observed that the detected causal relations are consistent using the proposed IG-based TE and the traditional TE. Thus, the proposed method ensures the accuracy of the detection of causal relations in this industrial case study.

To distinguish the direct causal relations from the indirect ones, the IG-based DTEs were calculated. From the causal network in Figure 9, the intermediate or confounding variables can be identified from the triangle connection paths. Then, the IG-based DTE should be calculated to determine whether the causal relation is direct or through such indirect paths via intermediate or confounding variables. For instance, there were two pathways from $T_{1}$ to $F_{4}$, i.e., direct or indirect through $T_{2}$. Then, the DTE from $T_{1}$ to $F_{4}$ based on $T_{2}$ was calculated and found to be smaller than the threshold; thus, the causal relation from $T_{1}$ to $F_{4}$ was determined to be indirect and the direct pathway from $T_{1}$ to $F_{4}$ was removed. It should be noticed that it was manually decided for which pairs of variables the IG-based DTE should be calculated. This is where the limitation of the proposed method lies. If the studied system is large and consists of many nodes or processes, making this decision is not easy. Thus, a way to automatically identify such triangle connection paths and, thus, determine intermediate conditioning variables is needed. A potential effective solution can refer to the algorithm in [50], which provides a practical way in large networks.

**Figure 10:**
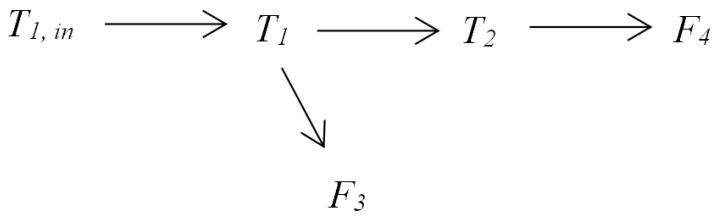
The direct information flow pathways for the two-tank system.

Table 7 provides the IG-based DTEs and the corresponding thresholds for different pairs of variables. As a result, the causal map containing only direct causal relations is obtained and shown in Figure 10. According to the obtained causal map, it can clearly see that $F_{1,in}$ is the the root cause variable of oscillation. The conclusion is consistent with the actual situation.

Next, the calculation time of the proposed method was compared with the traditional TE. The TEs between the five variables were calculated. The total calculation time of each method is shown in Table 8. It can be seen that the calculation time of the proposed IG-based TE method was reduced by 97.3% compared to the traditional TE. Thus, using the proposed method, it can quickly detect the causal relations between the candidate variables and ultimately find out the root cause of the fault. Compared with the traditional TE, the proposed method greatly improves the calculation efficiency and provides a basis for quickly locating the root cause of the fault.

The time calculated by the IG-based TE, including the time required for delay estimation and information granulation, is shown in detail in Table 9. It can be seen from the table that the delay estimation and information granulation before calculating TE takes little time. Compared with traditional TE, the calculation time is greatly reduced.

## 5. Conclusions

This proposes an improved method for causality inference based on transfer entropy and information granulation. Motivated by the problems accounting for the high computational complexity, a new framework is designed to integrate the information granulation as a critical preceding step to compress data, such that the abstracted representative features are obtained and used in TE calculation. The accuracy of the result is mainly affected by the length of the window size in information granulations. Thus, a window-length determination method is proposed based on delay estimation. Both a numerical case and an industrial case are presented to demonstrate the efficacy of the proposed method. According to the results, the proposed method is capable of detecting the causal relations correctly and promptly. In the numerical and industrial case studies, the proposed method uses only 11.8% and 2.7% of the calculation time of the traditional TE, respectively. Compared to the original TE, the proposed method shows significantly better computational efficiency, making it more appropriate in real-time applications for root cause analysis.

It should also be noticed that properly compressing the time series via granulation is critical to the correct estimate of the first-order TE, and this relies on the determination of the window length, which is set as the time delay between two time series. This paper assumes that the history of the target time series *y* should be no more than the delay between *x* and *y*, such that the data can be properly compressed and both the histories of *x* and *y* can be fully included in the estimate of the first-order TE. However, it is possible that the relevant history of the target time series *y* is much larger than the delay in real cases. According to the literature [46], failing to include the relevant history of the target time series can lead to a spurious overestimation of the TE. This is a problem worthy of deep investigation and which can be considered in future work to for a better solution to obtain a more accurate estimate of transfer entropy.

## Figures and Tables

**Table 1:** The value of TE versus the orders.

	IG-Based TE (x→y)
k=l=1	2.10
k=l=2	1.92
k=l=3	1.99
k=l=4	2.01
k=l=5	1.97

**Table 2:** IG-based TEs and thresholds (in the brackets).

	X	Y	Z
X		0.72 (0.34)	0.99 (0.30)
Y	0		0.22 (0.41)
Z	0	2.56 (0.51)	

**Table 3:** TEs and thresholds (in the brackets).

	X	Y	Z
X		1.57 (0.31)	1.44 (0.15)
Y	0.07 (0.20)		0.05 (0.16)
Z	0.12 (0.25)	2.60 (0.36)	

**Table 4:** Calculation time using the traditional TE and the IG-based TE.

	Traditional TE	IG-Based TE
Calculation time	3.4 s	0.4 s

**Table 5:** IG-based TEs and thresholds (in the brackets).

	$F_{1,\mathrm{in}}$	$T_{1}$	$T_{2}$	$F_{3}$	$F_{4}$
$F_{1,in}$		1.80 (0.24)	0.82 (0.20)	1.95 (0.21)	1.47 (0.24)
$T_{1}$	0.03 (0.16)		1.10 (0.21)	1.16 (0.26)	1.82 (0.25)
$T_{2}$	0.11 (0.21)	0		0.53 (0.15)	0.61 (0.20)
$F_{3}$	0	0.08 (0.24)	0.85 (0.15)		1.93 (0.20)
$F_{4}$	0	0.04 (0.21)	0.14 (0.26)	1.04 (0.16)	

**Table 6:** TEs and thresholds (in the brackets).

	$F_{1,\mathrm{in}}$	$T_{1}$	$T_{2}$	$F_{3}$	$F_{4}$
$F_{1,in}$		1.05 (0.24)	0.88 (0.36)	1.08 (0.18)	0.90 (0.25)
$T_{1}$	0.03 (0.16)		0.80 (0.43)	1.04 (0.39)	0.72 (0.30)
$T_{2}$	0.09 (0.20)	0		0.98 (0.48)	0.78 (0.29)
$F_{3}$	0	0.08 (0.16)	0.88 (0.35)		0.94 (0.24)
$F_{4}$	0	0.04 (0.18)	0.14 (0.26)	1.05 (0.40)	

**Table 7:** IG-based DTEs and thresholds.

From *x* to *y*	Intermediate Variables	IG-Based DTE	Thresholds
$T_{1,in}\to T_{2}$	$T_{1}$	0.08	0.20
$T_{1,in}\to F_{3}$	$T_{1}$	0.11	0.21
$T_{1,in}\to F_{4}$	$T_{1}$	0.05	0.24
$T_{1,in}\to F_{4}$	$T_{2}$	0.05	0.24
$T_{1}\to F_{4}$	$T_{2}$	0.13	0.25
$T_{2}\to F_{3}$	$T_{1}$	0.06	0.15
$F_{3}\to T_{2}$	$T_{1}$	0.05	0.15
$F_{3}\to F_{4}$	$T_{1,in}$	0.04	0.20
$F_{4}\to F_{3}$	$T_{1,in}$	0	

**Table 8:** Calculation time using the traditional TE and the IG-based TE.

	Traditional TE	IG-Based TE
Calculation time	76.8 s	2.1 s

**Table 9:** Detailed calculation time using the IG-based TE.

	Delay Estimation	Calculation of IG	Calculation of TE
Calculation time	1.1 s	0.2 s	0.8 s

## Data Availability

Not applicable.

## References

[1] Yang F., Duan P., Shah S.L., Chen T. (2014). Capturing Connectivity and Causality in Complex Industrial Processes.

[2] Jiang H., Patwardhan R., Shah S.L. (2009). Root cause diagnosis of plant-wide oscillations using the concept of adjacency matrix. J. Process Control..

[3] Yang F., Xiao D., Shah S.L. (2013). Signed directed graph-based hierarchical modelling and fault propagation analysis for large-scale systems. IET Control Theory Appl..

[4] Bauer M., Thornhill N.F. (2008). A practical method for identifying the propagation path of plant-wide disturbances. J. Process Control.

[5] Chen Q., Lang X., Lu S., ur Rehman N., Xie L., Su H. (2021). Detection and root cause analysis of multiple plant-wide oscillations using multivariate nonlinear chirp mode decomposition and multivariate Granger causality. Comput. Chem. Eng..

[6] Schreiber T. (2000). Measuring information transfer. Phys. Rev. Lett..

[7] Meng Q.Q., Zhu Q.X., Gao H.H., He Y.L., Xu Y. (2019). A novel scoring function based on family transfer entropy for Bayesian networks learning and its application to industrial alarm systems. J. Process Control.

[8] Raveendran R., Huang B. (2018). Variational Bayesian approach for causality and contemporaneous correlation features inference in industrial process data. IEEE Trans. Cybern..

[9] Yang F., Xiao D. (2012). Progress in root cause and fault propagation analysis of large-scale industrial processes. J. Control Sci. Eng..

[10] Duan P., Chen T., Shah S.L., Yang F. (2014). Methods for root cause diagnosis of plant-wide oscillations. AIChE J..

[11] Lindner B., Auret L., Bauer M., Groenewald J.W. (2019). Comparative analysis of Granger causality and transfer entropy to present a decision flow for the application of oscillation diagnosis. J. Process Control.

[12] Landman R., Jämsä-Jounela S.L. (2016). Hybrid approach to casual analysis on a complex industrial system based on transfer entropy in conjunction with process connectivity information. Control Eng. Pract..

[13] Mehler D.M.A., Kording K.P. (2018). The lure of misleading causal statements in functional connectivity research. arXiv.

[14] Lizier J.T., Prokopenko M. (2010). Differentiating information transfer and causal effect. Eur. Phys. J. B.

[15] Bauer M., Cox J.W., Caveness M.H., Downs J.J., Thornhill N.F. (2006). Finding the direction of disturbance propagation in a chemical process using transfer entropy. IEEE Trans. Control Syst. Technol..

[16] Jiao J., Zhen W., Zhu W., Wang G. (2020). Quality-related root cause diagnosis based on orthogonal kernel principal component regression and transfer entropy. IEEE Trans. Ind. Inform..

[17] Kugiumtzis D. (2013). Partial transfer entropy on rank vectors. Eur. Phys. J. Spec. Top..

[18] Duan P., Yang F., Chen T., Shah S.L. (2013). Direct causality detection via the transfer entropy approach. IEEE Trans. Control Syst. Technol..

[19] Duan P., Yang F., Shah S.L., Chen T. (2015). Transfer zero-entropy and its application for capturing cause and effect relationship between variables. IEEE Trans. Control Syst. Technol..

[20] Staniek M., Lehnertz K. (2008). Symbolic transfer entropy. Phys. Rev. Lett..

[21] Guo C., Yang F., Yu W. (2015). A causality capturing method for diagnosis based on transfer entropy by analyzing trends of time series. IFAC-PapersOnLine.

[22] Camacho M., Romeu A., Ruiz-Marin M. (2021). Symbolic transfer entropy test for causality in longitudinal data. Econ. Model..

[23] Rashidi B., Singh D.S., Zhao Q. (2018). Data-driven root-cause fault diagnosis for multivariate non-linear processes. Control Eng. Pract..

[24] Hu W., Wang J., Chen T., Shah S.L. (2017). Cause-effect analysis of industrial alarm variables using transfer entropies. Control Eng. Pract..

[25] Luo Y., Gopaluni B., Xu Y., Cao L., Zhu Q.X. (2020). A novel approach to alarm causality analysis using active dynamic transfer entropy. Ind. Eng. Chem. Res..

[26] Lindner B., Auret L., Bauer M. (2019). A systematic workflow for oscillation diagnosis using transfer entropy. IEEE Trans. Control Syst. Technol..

[27] Naghoosi E., Huang B., Domlan E., Kadali R. (2013). Information transfer methods in causality analysis of process variables with an industrial application. J. Process Control.

[28] Shannon C.E. (2001). A mathematical theory of communication. ACM SIGMOBILE Mob. Comput. Commun. Rev..

[29] Vicente R., Wibral M., Lindner M., Pipa G. (2011). Transfer entropy—A model-free measure of effective connectivity for the neurosciences. J. Comput. Neurosci..

[30] Barnett L., Seth A.K. (2017). Detectability of Granger causality for subsampled continuous-time neurophysiological processes. J. Neurosci. Methods.

[31] Smirnov D.A. (2013). Spurious causalities with transfer entropy. Phys. Rev. E.

[32] Guo Z., McClelland V.M., Simeone O., Mills K.R., Cvetkovic Z. (2021). Multiscale Wavelet Transfer Entropy with Application to Corticomuscular Coupling Analysis. IEEE Trans. Biomed. Eng..

[33] Florin E., Gross J., Pfeifer J., Fink G.R., Timmermann L. (2010). The effect of filtering on Granger causality based multivariate causality measures. Neuroimage.

[34] Faes L., Nollo G., Stramaglia S., Marinazzo D. (2017). Multiscale granger causality. Phys. Rev. E.

[35] Bossomaier T., Barnett L., Harré M., Lizier J.T. (2016). Transfer entropy. An Introduction to Transfer Entropy.

[36] Du S., Wu M., Chen L., Hu J., Jin L., Cao W., Pedrycz W. (2020). Operating mode recognition based on fluctuation interval prediction for iron ore sintering process. IEEE/ASME Trans. Mechatron..

[37] Lu W., Pedrycz W., Liu X., Yang J., Li P. (2014). The modeling of time series based on fuzzy information granules. Expert Syst. Appl..

[38] Guo H., Wang L., Liu X., Pedrycz W. (2020). Information granulation-based fuzzy clustering of time series. IEEE Trans. Cybern..

[39] Wang W., Pedrycz W., Liu X. (2015). Time series long-term forecasting model based on information granules and fuzzy clustering. Eng. Appl. Artif. Intell..

[40] Zadeh L.A. (1979). Fuzzy sets and information granularity. Adv. Fuzzy Set Theory Appl..

[41] Yu F., Pedrycz W. (2009). The design of fuzzy information granules: Tradeoffs between specificity and experimental evidence. Appl. Soft Comput..

[42] Faes L., Nollo G., Porta A. (2011). Information-based detection of nonlinear Granger causality in multivariate processes via a nonuniform embedding technique. Phys. Rev. E.

[43] Wollstadt P., Lizier J.T., Vicente R., Finn C., Martinez-Zarzuela M., Mediano P., Novelli L., Wibral M. (2018). IDTxl: The Information Dynamics Toolkit xl: A Python package for the efficient analysis of multivariate information dynamics in networks. arXiv.

[44] Schreiber T., Schmitz A. (2000). Surrogate time series. Phys. D Nonlinear Phenom..

[45] Kantz H., Schreiber T. (2004). Nonlinear Time Series Analysis.

[46] Wibral M., Pampu N., Priesemann V., Siebenhühner F., Seiwert H., Lindner M., Lizier J.T., Vicente R. (2013). Measuring information-transfer delays. PLoS ONE.

[47] Wollstadt P., Sellers K.K., Rudelt L., Priesemann V., Hutt A., Fröhlich F., Wibral M. (2017). Breakdown of local information processing may underlie isoflurane anesthesia effects. PLoS Comput. Biol..

[48] Lindner B., Auret L., Bauer M. (2017). Investigating the impact of perturbations in chemical processes on data-based causality analysis. Part 1: Defining desired performance of causality analysis techniques. IFAC-PapersOnLine.

[49] Lindner B., Auret L., Bauer M. (2017). Investigating the impact of perturbations in chemical processes on data-based causality analysis. part 2: Testing granger causality and transfer entropy. IFAC-PapersOnLine.

[50] Wollstadt P., Meyer U., Wibral M. (2015). A graph algorithmic approach to separate direct from indirect neural interactions. PLoS ONE.

